# Role of Negative Suction Therapy and Platelet-Rich Plasma in the Management of Delayed Post-operative Wound Healing: A Case Report

**DOI:** 10.7759/cureus.55553

**Published:** 2024-03-05

**Authors:** Abhiram Awasthi, Aditya Kekatpure, Aashay Kekatpure, Shivshankar Jadhav

**Affiliations:** 1 Department of Orthopaedics, Jawaharlal Nehru Medical College, Datta Meghe Institute of Medical Sciences, Wardha, IND; 2 Department of Orthopaedic Surgery, Jawaharlal Nehru Medical College, Datta Meghe Institute of Medical Sciences, Wardha, IND

**Keywords:** advanced wound care, postoperative wounds, platelet-rich plasma (prp), vacuum assisted closure (vac), tibial pilon

## Abstract

Tibial Pilon fractures are rare yet devastating injuries. To classify these fractures, the Arbeitsgemeinschaft für Osteosynthesefragen (AO) classification system is the most commonly used method. Out of all the different types, type C fractures are the most difficult to manage because the enormous energy involved in creating this type of injury typically severely destroys the soft tissue surrounding the fracture zone. As a result, long-term outcomes are frequently poor, and proper initial primary care is critical. Pilon fractures are injuries that are difficult to manage, considering the poor soft tissue envelope. These injuries often are associated with delayed wound healing and require staged management. Additional methods of treating the soft tissue envelope are currently being investigated and have shown promising results for the future. We share our experience in the management of AO type 43C3 grade I compound distal tibia fibular fracture with post-operative wound dehiscence, successfully managed with vacuum-assisted closure (VAC) and platelet-rich plasma (PRP) therapy.

## Introduction

Pilon fractures, also known as tibial plafond fractures, may be caused by low- to high-energy axial loading traumas. This very uncommon injury, which accounts for 10% of all lower extremity fractures, mainly affects people in their 30s and 40s who have fallen from a height or been in a car accident [1]. Males (57%) are more likely than females (65%) to suffer from Pilon fractures [2]. The age distribution of Pilon fractures is bimodal, with a peak incidence between the ages of 25 and 50 [2]. Most weight-bearing part of the ankle joint is composed of the distal tibia. Pilon fractures are fractures that involve this weight-bearing part. These fractures are caused by high-velocity trauma with direct axial rotational force.

The Arbeitsgemeinschaft für Osteosynthesefragen (AO) classification system describes these fractures in three categories. Type A fractures are extra-articular distal tibia fractures. They are subdivided into A1, A2, and A3 based on the comminution of the metaphysis. Type B fractures are partial articular fractures where a part of the articular surface remains in contact with the shaft of the tibial shaft. They are subdivided into B1, B2, and B3 based on the quantity of articular impaction and comminution. Type C fractures are complete metaphyseal fractures combined with the articular surface involvement. They are subdivided into C1, C2, and C3 based on the articulation and metaphyseal comminution. Type C fractures are a complex type of Pilon fractures [3,4]. High-intensity Pilon fractures are most commonly associated with fibular fractures and complex soft tissue injury [5].

Surgical treatment of these complex fractures is challenging as they are frequently associated with high infection rates, post-traumatic arthritis of the ankle joint, and soft tissue necrosis [6]. Many treatment modalities have been described to overcome these complications and accurately reduce the fracture, such as open reduction and internal fixation, external fixation, percutaneous plating, and a two-staged protocol involving external fixation and then open reduction and internal fixation with plating. The benefit of this two-stage procedure is that it allows the soft tissue injury to recover, reduces the risk of skin necrosis, and prevents wound infections [7,8].

Following surgeries for these types of fractures, great attention must be given to soft tissue coverage and wound complications. The area of the tibial plafond has less soft tissue envelope. If there is a risk of skin necrosis or soft tissue necrosis after the second stage of open reduction and internal fixation, complete debridement of the necrosed region followed by platelet-rich plasma (PRP) infiltrations and vacuum-assisted closure (VAC) of the wound can aid in the final proper management of the injury.

## Case presentation

**Figure 1:**
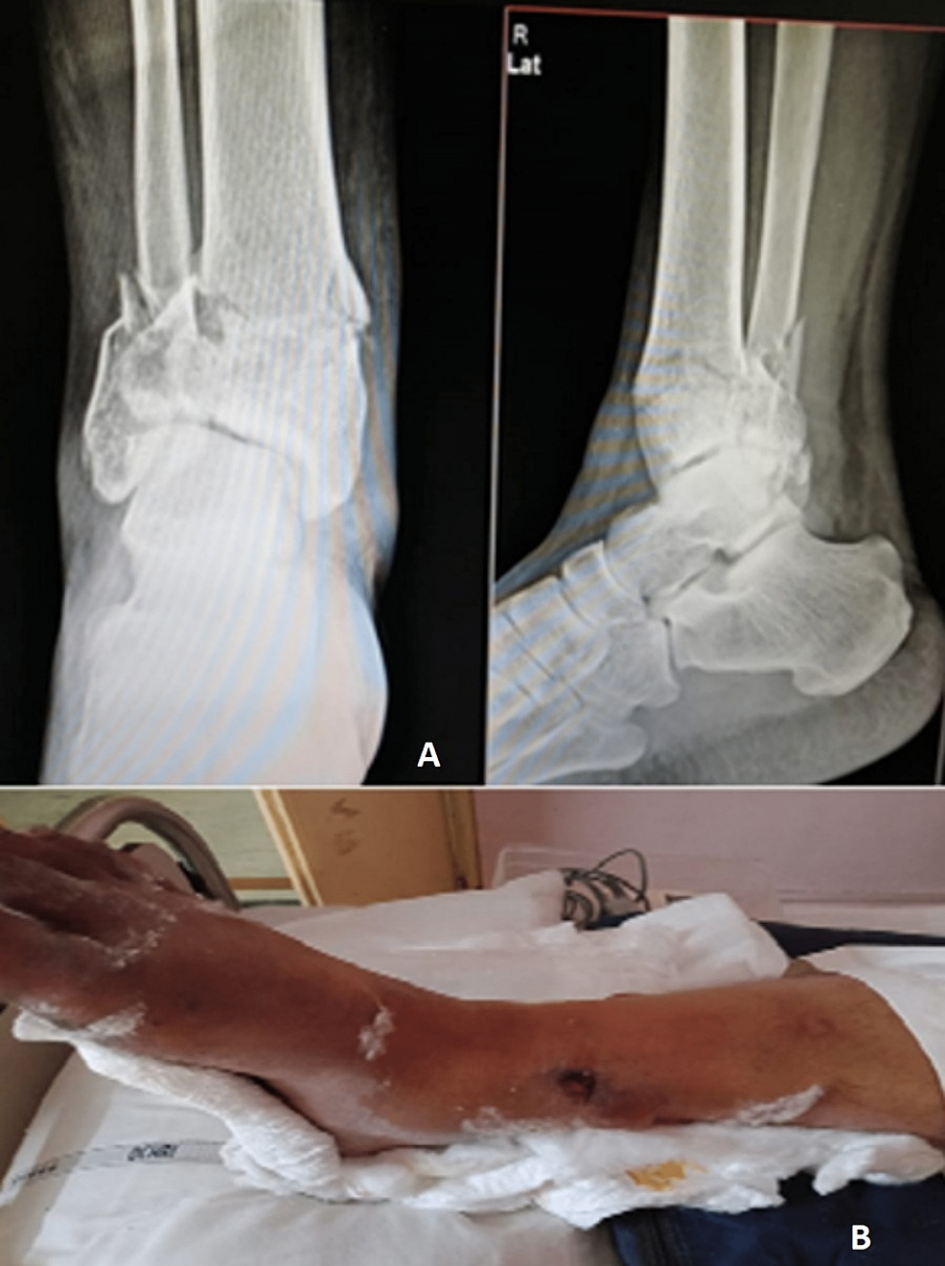
Pre-operative X-rays and clinical image A: Pre-operative X-rays in anteroposterior and lateral views B: Pre-operative clinical image

A 65-year-old male presented to the outpatient department (OPD) with pain and swelling over his right ankle with an exposed wound over the anterior aspect of the distal leg and bone. X-rays were done, and he was diagnosed with a compound grade 3A Pilon fractured right leg. At the OPD, thorough wound debridement was performed, and then an above-knee slab was given. Basic investigations of the patient were performed, and a computed tomography (CT) scan of the right ankle was done (Figure 1A).

**Figure 2:**
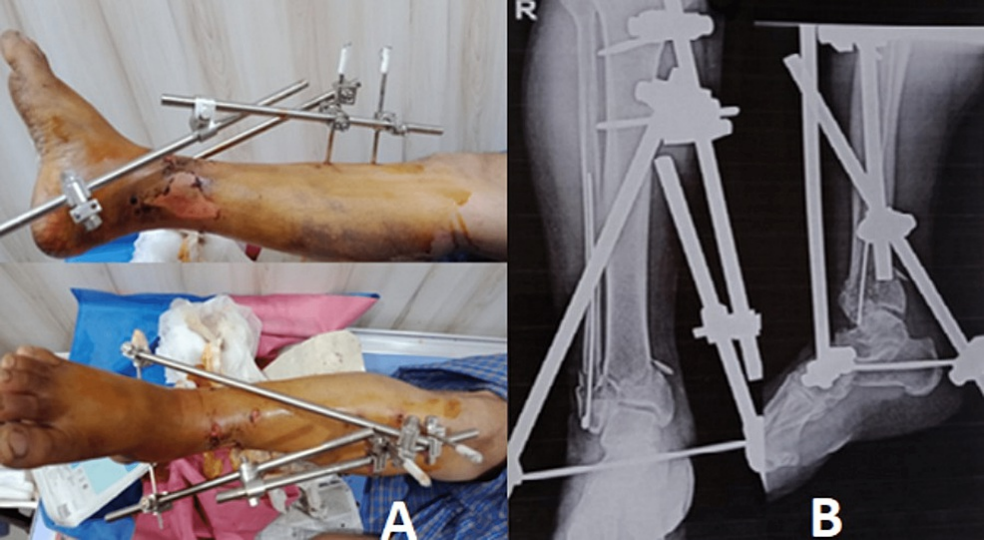
Immediate post-operative clinical image and X-rays (Step 1) A: Clinical image of the delta fixation B: Post-operative X-rays in anteroposterior and lateral views

CT scans of the patient helped classify the fracture as AO type C1. The patient was taken to the operation theatre, where the fracture site was thoroughly explored under spinal anaesthesia. The fracture site was found to be grossly comminuted. Firstly, the fibula was stabilised and the fractured fibula was managed with rush nail insertion. Then, fracture fragments of the tibia were aligned and the complete ankle joint was stabilised by delta external fixation (Figure 2).

**Figure 3:**
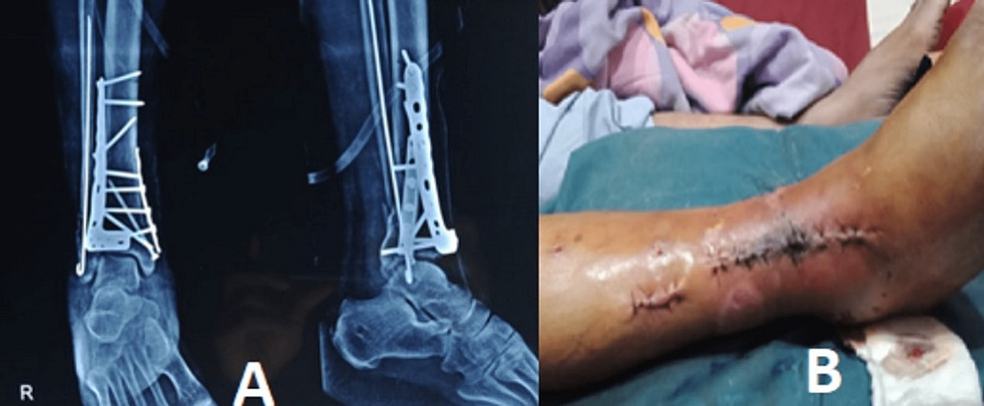
Open reduction and internal fixation X-rays and clinical image of the wound (Step 2) A: Post-operative X-rays in anteroposterior and lateral views B: Clinical image of post-operative day two surgical site

The patient was closely monitored for a few days with regular wound dressings. After 15 days of continuous monitoring of the patient's condition, the compound wound was healed, and the soft tissue and swelling subsided. The patient was prepared for the next step, which was the removal of the external fixator, open reduction, and internal fixation. The patient was taken to the operation theatre, and with an anterolateral approach to the ankle, bi-columnar plating was performed with a distal tibia periarticular locking plate. For stability, two cancellous screws were also inserted from the medial malleolus of the tibia. Primary closure of the incision was done, and a below-knee slab was applied (Figure 3).

**Figure 4:**
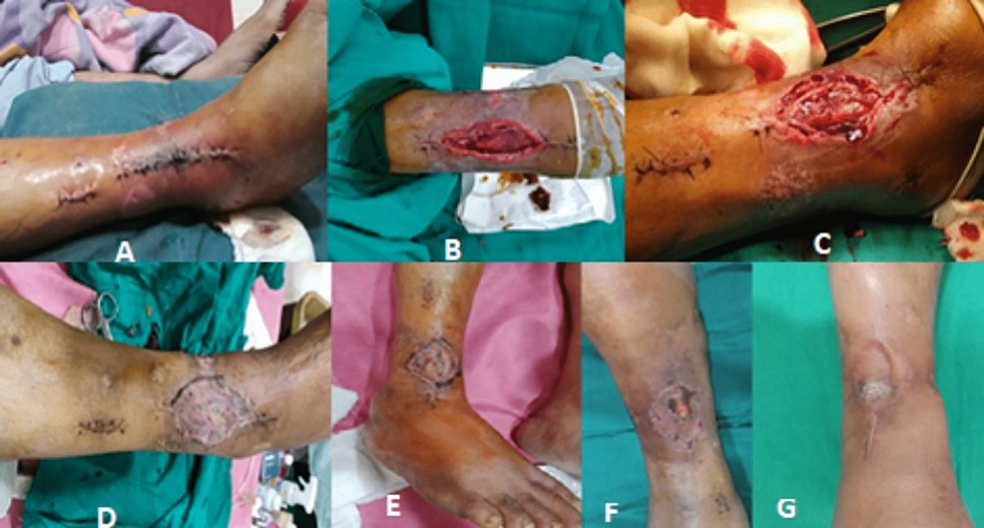
Series of clinical images of wound management with VAC and PRP infiltration followed by skin grafting A: Wound after five days B: Intraoperative debridement and VAC application C: PRP infiltration over the wound every fourth day D: Post-operative day 10 with skin grafting done E: Post-operative day 14 F: Post-operative day 18 G: Post-operative day 22 with completely healed wound VAC: Vacuum-assisted closure; PRP: Platelet-rich plasma

After seven days of VAC application, wound size was significantly reduced. The wound was afterward managed with a series of PRP infiltrations. The PRP infiltrations were done twice weekly in four-day intervals with a 22G needle along the wound margins of the skin flap until the wound was completely healed. It was done using the STARS therapy protocol [9]. The wound healed after four sessions of PRP infiltrations, leaving just a tiny scar (Figure 4).

The right lower limb was immobilized on a below-knee slab, and the patient was discharged. After 1.5 months, the slab was opened and X-rays were taken. The X-ray results showed good fracture healing. The slab was removed, and ankle range of movements physiotherapy exercises were started. After six months of vigorous physiotherapy and rehabilitation, the patient was relieved and began his daily work. The patient was pain-free, and the ankle movements were complete and painless, with no signs of wound infection or ankle arthritis.

At the last follow-up after 18 months, the American Orthopaedic Foot and Ankle Society (AOFAS) score of the patient was 100 points or 100% with no restriction in daily activities. The patient was completely relieved of pain and achieved a full range of movements in dorsiflexion and plantarflexion. The patient also achieved complete radiological healing at the end of 18 months.

## Discussion

High-energy trauma with rotational force usually causes Pilon fractures, which are associated with severely injured soft tissue. According to Barei et al. [5], Pilon fractures combined with fractures of the fibula are more severe than those without fractures. Anatomical reduction of the fracture without surgical intervention is challenging in these cases [10]. The clinical picture of the soft tissue injury is delayed in these fractures, and peak inflammation and oedema usually occur three to five days after the injury. Surgical intervention further increases the mild tissue injury, and if open reduction and internal fixation are performed first, then the tension at the incision site increases. It finally causes a reduced blood supply to the soft tissue. As a result, wound infections and skin necrosis of the suture site commonly occur. Therefore, it is essential to choose a perfect timing to operate.

Bourne et al. [11] reported a 13% risk of deep infection in high-energy injuries after open reduction and internal fixation of the distal tibia. After three attempts at arthrodesis, the limb was amputated. In the most extensive series of Pilon fractures treated surgically to date, Dillin and Slabaugh [12] observed devastating consequences, including a 36% incidence of skin slough and a 55% risk of infection when the fracture had unstable fixation. In 1992, McFerran et al. [13] noted a wound disintegration rate of 24% and an infection rate of 17%, with one patient needing an amputation. Teeny and Wiss [14] pointed out that a study of patients with high-energy fractures found skin slough in 27% of cases and a 37% infection rate.

A staged program for treating type C Pilon fractures was recently implemented to counter the complications. The initial step of the technique involves immediate bone traction and the use of an external fixator. Formal open reconstruction of the articular surface was accomplished in the second step. The incidence of severe soft tissue complications, such as soft tissue injury, skin necrosis, wound dehiscence, articular cartilage damage, and deep infection, was dramatically reduced with the strategy [15]. Ketz and Sanders [16] reported that after therapy with a staged protocol for treating type C Pilon fractures, 14 of their 16 patients (88%) had excellent results.

We used these two staged protocols for the management and got excellent results. Reducing soft tissue damage and the risk of deep infection in high-energy Pilon fractures remains a therapeutic challenge. VAC technique is a new wound-healing technique. It is particularly well suited for the treatment of complicated wounds as compared to other standard drainage methods. According to Miller et al. [17], the negative pressure environment improves subcutaneous blood flow, limits bacterial proliferation, and promotes granulation formation in the wound. Furthermore, clinical research has shown that combining VAC with debridement enhances wound healing, reduces hospitalisation duration, and improves patient satisfaction [18]. In this case, we combined VAC therapy with a series of PRP infiltrations, which gave excellent results.

Suppose the flap necrosis sets in or infections occur in the suture site. In that case, VAC is a suitable option as it significantly reduces the illness and provides a healthy environment for wound closure. VAC shows excellent results, and the healing time is also improved. After removing the VAC dressing, the remaining wound can be managed with a series of PRP infiltrations for the best results in a minimal time interval.

## Conclusions

As for orthopaedic surgeons, tibial Pilon fractures present an immense challenge though they are rare in routine practice. Possible risk factors need identification pre-operatively, which can be done by performing some investigations, such as CT scans and a complete blood profile that ensures successful treatment and outcomes. Tibial Pilon fractures need utmost care and importance, taking into particular consideration the fragile soft tissue envelope and the watershed area. Each fracture pattern is different, and choosing the right approach for each fracture pattern is necessary to visualise the fracture and anatomically reduce the articular surface. Modern surgical techniques and hardware have improved operative outcomes but still have a high overall complication rate. Also, operating surgeons face a complication after two staged surgical management of such fractures, which is post-operative wound dehiscence. Viable options for such complications include negative suction therapy, PRP infiltrations, and skin grafting, which gave good results in our patients.
